# The Forensic Restrictiveness Questionnaire: Development, Validation, and Revision

**DOI:** 10.3389/fpsyt.2019.00805

**Published:** 2019-11-15

**Authors:** Jack Tomlin, Birgit Völlm, Vivek Furtado, Vincent Egan, Peter Bartlett

**Affiliations:** ^1^Department of Forensic Psychiatry, University of Rostock, Rostock, Germany; ^2^Mental Health and Wellbeing, Warwick Medical School, University of Warwick, Coventry, United Kingdom; ^3^Centre for Family and Forensic Psychology, University of Nottingham, Nottingham, United Kingdom; ^4^School of Law and Institute of Mental Health, University of Nottingham, Nottingham, United Kingdom

**Keywords:** forensic, mental health, restrictive, autonomy, FRQ, forensic restrictiveness questionnaire, psychometric

## Abstract

**Introduction:** Forensic psychiatric care is often practiced in closed institutions. These highly regulated, secure, and prescriptive environments arguably reduce patient autonomy, self-expression, and personhood. Taken together these settings are restrictive as patients’ active participation in clinical, organizational, community, and personal life-worlds are curtailed. The consequences of patients’ experiences of restrictiveness have not been explored empirically. This study aimed to develop a psychometrically-valid measure of experiences of restrictiveness. This paper presents the development, validation, and revision of the Forensic Restrictiveness Questionnaire (FRQ).

**Methods:** In total, 235 patients recruited from low, medium, and high secure hospitals across England completed the FRQ. The dimensionality of the 56-item FRQ was tested using Principle Axis Factor Analysis and parallel analysis. Internal consistency was explored with Cronbach’s α. Ward climate (EssenCES) and quality of life (FQL-SV) questionnaires were completed by participants as indicators of convergent validity. Exploratory Factor Analysis (EFA) and Cronbach’s α guided the removal of items that did not scale adequately.

**Results:** The analysis indicated good psychometric properties. EFA revealed a unidimensional structure, suggesting a single latent factor. Convergent validity was confirmed as the FRQ was significantly negatively correlated with quality of life (Spearman’s *ρ* = −0.72) and ward climate (Spearman’s *ρ* = −0.61). Internal consistency was strong (α = 0.93). Forty-one items were removed from the pilot FRQ. The data indicate that a final 15-item FRQ is a valid and internally reliable measure.

**Conclusion:** The FRQ offers a novel and helpful method for clinicians and researchers to measure and explore forensic patients’ experiences of restrictiveness within secure hospitals.

## Introduction

Secure hospitals aim to provide a safe, therapeutic milieu in light of restrictive risk-averse policies and practices. The provision of forensic beds has been steadily increasing over the past decades in several European countries (1). A growing number of individuals are therefore placed within settings that have been described elsewhere as ‘total’, subject to prescriptive daily regimes (2, 3).

Efforts to provide mental health care in the least restrictive environment possible are recognized internationally. In Canada, the Ontario Court of Appeal has held that not criminally responsible dispositions must be the “least onerous and least restrictive” (Osawe (Re), 2015 ONCA 280). Across Europe, Salize et al., (4) found that, of the 15 European Union member states they investigated, 13 codified the notion of “less restrictive” facilities or medication into law. In the UK, policy and best practice guidelines that reference least restrictive practice are ubiquitous (4–11).

The prevalence of the least restrictive ideal across different stakeholders reflects historical trends. These trends involve movements away from large asylums and a recognition that patient autonomy and involvement in care should be maximized (12). These trends are captured in international human rights instruments, contemporary models of offender rehabilitation, and research into coercive and restrictive measures.

The Council of Europe describes a minimum standard of care and accommodation that centers that deprive individuals of their liberty (including forensic hospitals) need to meet. These provisions intend to safeguard individuals from arbitrary, disproportionate, and unjustified detention; facilitate patient individuality and expression; and maximize the exercise of agency in patients’ private lives. In Recommendation REC(2004)10, the Council of Europe makes plain that patients should receive care in the least restrictive environment possible (art. 8). This environment should:

*‘[…] provide each such person, taking into account his or her state of health and the need to protect the safety of others, with an environment and living conditions as close as possible to those of persons of similar age, gender and culture in the community. (* 13 *: art. 9)*.

Some forensic settings have begun to embrace elements of the recovery paradigm (14–16). The recovery paradigm prioritizes the role of individual agency. It emphasizes that individuals should play a role in planning their care, daily life and take responsibility for their actions. This empowerment is contingent on a notion of autonomy and the ability to act as an independent agent (17). The recovery paradigm therefore presupposes that individuals with mental disorders ought to take responsibility for, and through empowerment, self-determine their actions (18). The difficulties of fully implementing recovery principles in secure settings have been highlighted; however, recovery principles are being introduced in some sites (19).

Best practice in forensic care is moving away from highly restrictive coercive measures (20). Coercive measures such as restraint, seclusion, and forced medication can, *in extremis,* preclude patient autonomy entirely. Accordingly, the use and consequence of coercive measures has become the focus of much recent research (21–23). Best practices to reduce their use have been developed (e.g., in England and Wales: the Mental Health Safety Improvement Programme to Reduce Restrictive Practices developed by NHS Improvement and the Care Quality Commission). Studies consistently report patients feel coercive measures limit autonomy, violate human rights, disrespect and dehumanize them and leave them feeling ignored (24, 25). Kontio et al. (26) report that coercive measures undermined satisfaction in care, treatment adherence, and violated patient autonomy. Thus, coercive measures are highly restrictive and can lead to negative patient outcomes.

### Defining Restrictiveness From Patients’ Perspectives

Recent studies have explored patients’ experiences of the restrictiveness of secure care more broadly. Sustere and Tarpey (27) asked residents in an English medium secure unit whether the introduction of Least Restrictive Practices on their unit increased autonomy and recovery. They found that participants felt the Least Restrictive Practices culture led to more positive risk-taking, greater levels of responsibility, and less judgement from staff (27). When asked to describe restrictive practices, residents identified restrictions on social interactions, which made them feel isolated, and restrictions on their ability to take control over aspects of their care particularly in relation to risk management.

Hui (28) interviewed 28 patients residing within a high secure hospital in England. Residents described restrictive practices as encompassing close confinement with others, a lack of private space and having few personal belongings. They expressed feeling frustrated by confusing or unfair rules and regulations. They suggested the environment promoted dependence on others and described feeling physically and mentally confined.

Tomlin et al. (29) qualitatively investigated 18 patients’ experiences of restrictiveness in low, medium, and high secure settings. Building on the conceptual work of Sexton (30) we found that patients’ experiences of restrictions could be described as *severe* and *salient*. The *severity* of restrictions for patients depended on to what extent residents felt aspects of care affected their autonomy, sense of self, or existence as a human being. The *salience* of restrictions described how psychologically significant these were for patients; this significance marked the degree to which patients expected or were surprised by restrictions or if these clashed with patients’ sense of what was fair. Where these expectations clashed with reality, restrictions were more salient. These accounts suggest that restrictions experienced by patients are subjective, diverse, and encompass more than coercive measures typically defined.

The definition of restrictiveness used for this project was derived from qualitative interviews conducted with patients reported by Tomlin et al. (29). The definition of restrictiveness, taken from the aforementioned study and the wider literature, used to guide the development of the pilot FRQ in the present study was:

*Restrictivenessis the extent to which phenomena created, maintained or augmented directly or indirectly by forensic psychiatric care are subjectively experienced by a resident as infringing negatively upon their autonomy, self or personhood.*

## Rationale and Aims

Despite recent qualitative efforts to conceptualize restrictiveness from patients’ perspectives there currently exists no valid and reliable measure that has been developed from interviews with patients and psychometrically validated. The closest is a version of the Measuring Quality of Prison Life Questionnaire adapted for forensic psychiatric settings (aMQPL) (31). The authors combined the domains “Transparency of procedures and decisions,” ”Fairness,” and ”Respect” to measure perceived institutional restraint alongside psychopathological symptoms and suicidal ideation across 130 patients in German forensic hospitals. Further instruments on involuntary admission (32) and coercive measures (33) exist, but these focus on procedural aspects of care or are event-related.

The present study sought to develop and validate the Forensic Restrictiveness Questionnaire (FRQ). This is a measure of restrictiveness that captures patient perspectives; considers myriad phenomena identified as restrictive by patients; and measures restrictiveness as a state, amenable to change and intervention over time. The FRQ permits measurement of whether efforts to implement least restrictive practices are experienced as such from a patient perspective. A valid instrument permits comparison of scores across groups, and associations with outcomes such as: recovery, aggressive incidents, recidivism, quality of life, and so forth.

The aims of this study were:

1. To develop and pilot the FRQ.
2. To assess the psychometric properties of the pilot FRQ.
3. To revise the FRQ in light of this.

## Methods

### Design

This study was observational and cross-sectional. The development of the FRQ followed the framework for developing, validating, and revising questionnaires forth by Adcock and Collier (34) and developed for a mixed-methods research design by Luyt (35). This framework comprises three stages: conceptualization, operationalization, and scoring cases. In the conceptualization stage, a “background concept” was defined and developed into a “systematized concept.” A literature review to develop the background concept was presented in Tomlin et al. (3).

Qualitative interviews with N = 18 patients in low, medium, and high secure settings in England were conducted and Thematically Analysed to generate the systematized concept (29). Items on the FRQ were derived from interviews. Patients described: restrictions on their sense of self given their treatment in forensic hospitals, the limited range and meaningfulness of activities, the prospects of reintegration into the community, the pathologization by staff of patient behaviors, reduced possibilities to exercise choice, and relationships with others inside and outside the hospital as restrictive and restricted (29).

In the second stage the systematized concept was operationalized into a pool of items that captured restrictiveness as a latent construct. These 80 items were discussed in the research team and 65 items were sent to a panel of five experts to assess their face validity. Participants had expertise in clinical forensic psychiatry; academic research on repression in Young Offender Institutions, and ward atmosphere in secure hospitals; national mental health policy development; and speech and language therapy in secure settings. Respondents were asked to what extent: a) each item reflected restrictiveness so defined; and b) whether each item would likely be interpretable by the target population. Following this, 56 items were included in the pilot FRQ.

The third stage involved the piloting and validation of the psychometric properties of the FRQ. Scale content (content validity), internal structure (dimensionality), associations amongst scores, and other variables (convergent validity) were investigated as measures of “construct validity” (36). Reliability (internal consistency) was also examined (37–39). Finally, the FRQ was revised in light of the piloting phase and psychometric properties.

### Setting

The study took place in secure forensic hospitals spread across England. These hospitals provide treatment to individuals detained under the Mental Health Act, 1983. Participants came from low, medium, and high secure hospitals in 16 National Health Service (NHS) Trusts (organizational units that serve a particular geographical area or medical specialty).

### Participants

The sampling frame comprised the forensic inpatient population of the 16 NHS Trusts. These Trusts were involved with the help of the NIHR Clinical Research Network. Sampling proceeded as primarily non-probabilistic and convenient but with some purposiveness (40–42). Wards providing care at different stages of recovery (e.g., rehabilitation, treatment, and admission) and hospitals of all levels of security were included. A range of hospitals and wards that provided care for different populations according to gender or diagnosis were approached. Most forensic in-patients were eligible for the study. The inclusion criteria were: sufficient grasp of the English language (or with use of translator if requested), and capacity to consent and participate; exclusion criteria were: a primary diagnosis of a learning disability, patients that were too unwell to participate (asserted by patient or staff), or under the age of 18.

## Instruments

### Essen Climate Evaluation Schema (EssenCES)

The EssenCES patient-version is a self-report measure of ward climate (43). This scale was initially designed in German and subsequently translated into English. The scale is composed of 15 items measured on five-point Likert scales across three domains. The domains include therapeutic hold (TH), experienced safety (ES), and patient cohesion (PC).

It demonstrated strong psychometric properties in its initial validation in a German sample (N = 327) (43). Principle Components Analysis supported the above domains, indicating good content validity. Internal consistency was demonstrated for each domain (Cronbach’s Alpha (α) = 0.87, 0.79, and.80 for TH, ES, and PC, respectively. The EssenCES has been validated in an English secure setting (44). A higher score indicates greater satisfaction with ward climate.

### Forensic Inpatient Quality of Life Questionnaire - Short Version (FQL-SV)

Patient quality of life was measured with the short version of the Forensic Inpatient Quality of Life Questionnaire - Short Version (FQL-SV; 45, 46). This scale was developed in The Netherlands and translated into English by its authors. The FQL-SV is comprised of 20 items. It asks patients about a range of topics including leave, safety, food, personal hygiene, sexuality, and relationships with other residents.

It has demonstrated good psychometric properties in a Dutch sample (45). Internal consistency was good (α = .79). Convergent validity was demonstrated as the FQL-SV correlated significantly with the World Health Organization’s WHOQOL-Bref QoL measure and the EssenCES measure of ward climate. A higher score indicates greater satisfaction with quality of life. The FQL-SV has a visual analogue scale from 0–100. This was recoded into 10 data points (1–3, 5–10). This recoding was necessary as in several participating sites printing issues meant the VAS line was 96 mm long. Patients that marked 96 on these scales are consequently comparable to those that marked 100 on the complete scales.

### The Pilot Forensic Restrictiveness Questionnaire (FRQ)

The pilot FRQ had 56 items each with a five-point Likert scale. Responses included “strongly disagree” through “strongly agree”. A Not Applicable option was also offered. The pilot FRQ included two ancillary questions asking: “How restricted do you feel in general?” and “Has anything very hard/difficult/hurtful happened to you in the last week?”. A higher score indicates a greater amount of experienced restrictiveness. Examples of items include: “The hospital helps me if I want to contact people outside,” “I am given enough information about my care,” “Staff stop me doing what I want,” and “The restrictions on the ward make sense.” Some items were reverse-coded to mitigated fatigue bias in responses. Space was allocated for patient feedback on the pilot FRQ.

### Procedure

The project was presented to patients and staff at ward community meetings. Interested patients could approach a member of the research team directly or by indicating their interest to staff. Patients were given information sheets and the project was explained to them. Patients were given at least 24 h to reconsider participation. All participants gave written consent.

Data on participants’ legal, clinical, and demographic profiles were collected. These data provided a descriptive account of participants depicted in Table and allowed analysis of significant differences between groups (to be published elsewhere). Data on age, gender, ethnicity, mental health diagnosis, index offence (if applicable), Mental Health Act (1983) section, and length of stay in current hospital were collected from patient notes by a member of the research team and grouped by the first author.

### Ethical Approval

Ethical approval was granted by the Leicestershire South Research Ethics Committee. Administrative approval was granted by the Health Research Authority of the NHS. The study reference code was: 17/EM/0159.

### Data Analysis

The analysis was conducted with STATA v.15. SPSS v.24 was used to impute missing data. Non-parametric alternatives were used where appropriate. Significance levels were set to p = 0.001 unless indicated.

#### Initial Item Removal

Prior to Factor Analysis items were removed if they had high collinearity with another item (Spearman’s *ρ* = > .0.7); had ceiling effects [>50% of responses fell on a single item and >80% were for agree or disagree (including the “Not Applicable’ option”)]; had Corrected Item-Total Correlation (CITC) scores <0.3; or where items were felt to be qualitatively redundant after piloting.

#### Factor Analysis

EFA was undertaken to explore the underlying structure of the FRQ (47). EFA is an iterative, data-driven approach that groups together variables that might then be hypothesized by the investigator to reflect respondents’ scores on a latent variable (47–50). Principle Axis Factoring was conducted with Oblique, PROMAX rotation as it was hypothesized resulting factors would be influenced by the latent construct of restrictiveness and would correlate (48). Items that loaded onto a factor <0.3 were considered weakly associated and were not considered for further analysis (47, 49, 50). Items were excluded from further analysis if they cross-loaded >0.3 onto two or more factors.

The decision to retain factors was based on several criteria: the Kaiser-Criterion rule of Eigenvalues >1.0; scree plot analysis; and parallel analysis (47). Parallel analysis based on the Monte-Carlo simulation technique was used with 10,000 repetitions. Observed factors with Eigenvalues greater than those generated in the parallel analysis were considered for retention, as this minimizes the generation of spurious factors due to chance association. Numerous models with different factorial solutions were computed before the most meaningful structure was arrived at.

#### Convergent Validity

Convergent validity explored the extent to which the pilot FRQ correlated in a hypothesized fashion with quality of life (FQL-SV) and ward climate (EssenCES). Spearman’s RHO was used as the FRQ and FQL-SV data were not normally distributed (49).

#### Reliability

Internal consistency is a measure of reliability and was investigated with Cronbach’s Alpha. An α > 0.7 was considered the minimum for a satisfactory score (38). Individual items with CITC scores <0.3 were considered not to measure the latent construct of restrictiveness and were removed.

#### Differences Between Groups

A Mann-Whitney U test was conducted to investigate whether participants who stated they experienced something very hard, difficult, or hurtful in the last week (an ancillary question on the FRQ) scored differently than those not reporting this. The Mann-Whitney U test was calculated as the data were non-parametric (49, 51).

#### Missing Data and Sampling Adequacy

Missing data represented 0.6% of all questionnaire data. Little’s test of missing completely at random indicated that data were missing at random: χ^2^(2686) = 2749.0, p = 0.194. The data were suitable for multiple imputation (52). Values were imputed with SPSS’s version 24 Automatic Imputation Method.

To assess the adequacy of the data for EFA the Kaiser-Meyer-Olkin (KMO) measure of sampling adequacy and Bartlett’s Test of Sphericity were calculated. KMO scores >0.7 suggest data are influenced by underlying factors (37). Bartlett’s Test with a significance value p < .05 indicates the overall item correlation matrix was significantly different from an identity matrix (52).

## Hypotheses

1. No hypothesis was put forward as to the dimensional structure of the pilot FRQ as this was exploratory.
2. The pilot FRQ would correlate negatively with both the FQL-SV and EssenCES.
3. The pilot FRQ would be internally consistent.

## Results

### Participants

In total, 241 patients were recruited. Data for six participants who did not complete at least one questionnaire were excluded. The following describes the largest participant groups; for complete data see **Table 1**. Participants were predominantly male (96%) and white (70%). Black and Caribbean participants comprised 16% of the sample. Mean participant age was 39 years (S.D. = 10.8; Min = 19 Max = 74). Median length of stay in current hospital was 19 months (Min = 1 Max = 277).

**Table 1:** Participants’ demographic, clinical, and legal profiles.

Variable		Frequency	%
Security Level			
Low		97	41
Medium		89	38
High		49	21
Total		235	100
Sex			
Male		225	96
Female		9	4
Total		218	100
Ethnicity			
White		160	70
Black/Caribbean		36	16
Asian		16	7
Mixed		13	6
Other		5	2
Total		230	100
Diagnosis			
F.6 Personality disorder		37	16
F.2 Mental illness		140	60
Mixed F.6 + F.2		20	9
Mixed F.2 + Other		16	7
Mixed F.6 + Other		5	2
Mixed F.6 + F.2 + Other		2	1
Other^1^		11	5
Undiagnosed		1	1
Total		232	100
MHA Section			
s. 3		45	19
s. 37		30	13
s. 37/41		100	43
s. 41(5)		6	3
s. 45(A)		6	3
s. 47/49		38	16
s. 36		1	1
s. 48/49		5	2
s. 38		1	1
Total		232	100
Index Offence			
Offences against the person		87	37
Offences against property		18	8
Sexual offences		23	10
Other^2^		41	18
Mixed		36	15
No offence		25	11
Did not disclose		1	1
Awaiting trial		2	1
Total		233	100
Age (years)	N 235	Mean (S.D.) 39.3 (10.8)	Min, Max 19, 74
LoS (months)	N 231	Median (Q1, Q3) 19 (9, 53)	Min, Max 1, 277

^1^Includes: F.3 Mood disorders, F.84 Autistic Spectrum Disorders, F.0 Organic Brain Disorders.

^2^Includes: Fraud, Arson, Possession of bladed article/offensive weapon, Threats to send explosives, Affray, Making explosives.

The majority of participants were given a primary diagnosis of a mental illness (60%). Individuals with a personality disorder as primary diagnosis constituted 16% of the sample. Respondents with a mixed diagnosis of MI and PD comprised (9%); and those with MI and/or PD and an “other” diagnosis comprised 10%. The “other” category (5%) included: organic brain disorders, mood disorders, and Autistic Spectrum Disorders.

The largest group of participants were on a Hospital Order with Restrictions (43%); one-fifth were on civil sections for treatment (19%); and 16% were Prison Transfers with Restrictions. The majority of index offences were offences against the person (37%), followed by sexual offences (10%), and offences against property (8%). A number of respondents had “mixed” offences, e.g., combination of offence-types (15%) and 18% had an offence categorized as “other”.

### Initial Item Reduction

Nine items were removed before EFA was conducted: one item for high (Spearman’s *ρ* = > 0.6) collinearity with three other items; four items for CITC scores <0.3; three items for ceiling effects; and one item was felt not to reflect restrictiveness for qualitative reasons. The remaining 47 items (N = 235) were suitable for factor analysis (KMO = 0.923; Bartlett’s Test of Sphericity *χ* *^2^*(1081) = 5177.7, p < .001). The participant to item ratio was 5:1.

### Factor Analysis

**Figure 1:**
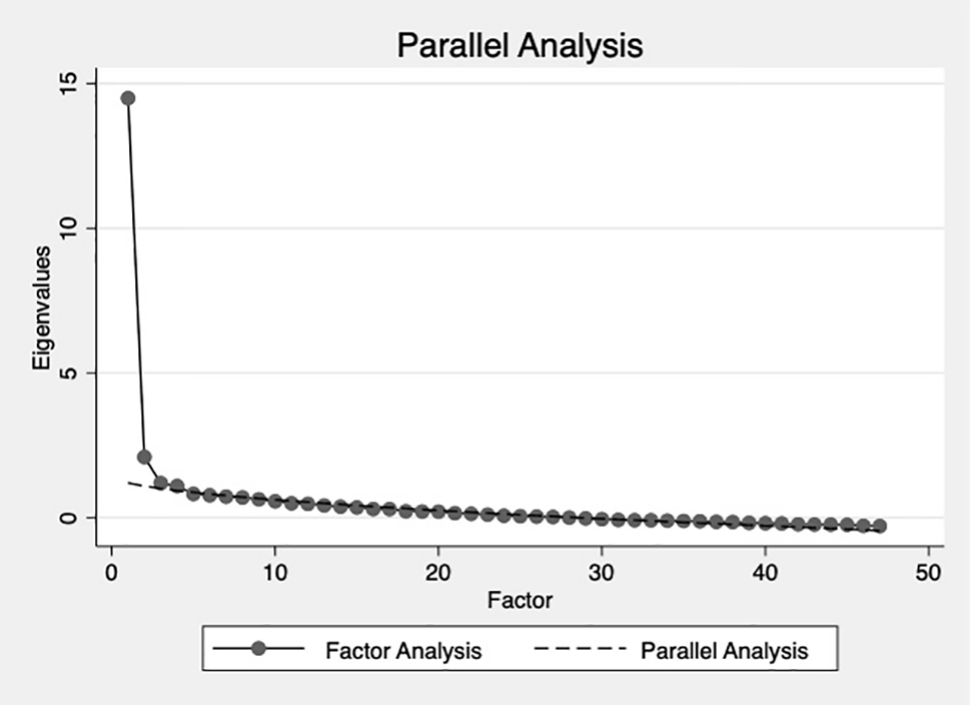
Parallel analysis and Principle Axis Factoring scree plot.

Principle Axis Factoring showed four factors with Eigenvalues greater than 1.0 (**Table 2**). These accounted for 78.8% of the variance. The first factor accounted for significantly more variance than the others. A scree plot supported this (**Figure 1**). Parallel Analysis using the Monte Carlo simulation technique with 10,000 iterations was then conducted to explore whether the four factors would occur by chance. This suggested the four observed factors were not likely to occur at random. This was consistent with the PAF results. Therefore, four factors were retained for extraction to iteratively explore the possible factorial structures.

**Table 2:** Principle axis factoring and parallel analysis values.

	Principle axis factoring		Parallel analysis
Factor	Eigenvalue	Variance	Eigenvalue
1	**14.49**	**0.61**	**1.20**
2	**2.09**	**0.09**	**1.10**
3	**1.19**	**0.05**	**1.01**
4	**1.09**	**0.05**	**0.94**
5	**0.82**	**0.03**	**0.87**
6	**0.77**	**0.03**	**0.82**
7	**0.73**	**0.03**	**0.76**
8	**0.70**	**0.03**	**0.71**
9	**0.65**	**0.03**	**0.66**
10	**0.57**	**0.02**	**0.62**

Bold denotes observed Eigenvalue greater than Parallel Analysis Eigenvalues.

Four factors were rotated using the PROMAX, oblique method (37). However, the fourth factor only contained three items of which all loaded onto at least one other factor >0.3. Further, the content of the factors did not group together in clinically or theoretically meaningful way. Factorial models with three and two factors were computed but items still did not group together in a meaningful way. For instance, the two-factor solution simply contained positively and negatively worded items. Given the lack of meaningful theoretical interpretation in the multi-factorial solutions, the Eigenvalues in **Table 2** and the scree plot in **Figure 1**, it was concluded that the underlying construct was unidimensional.

Items that loaded strongly onto this unidimensional structure were felt most reflective of restrictiveness. To keep the FRQ short, and in line with patient feedback the 15 highest loading items (0.62–0.72) on this single factor were retained. Item loadings and uniqueness scores (the amount of variance in each item not explained by the latent model) are presented in **Table 3**. The remaining items had a Flesch Reading Ease Score of 82.3, which corresponds to an average 11-year-old reading level (53).

**Table 3:** Item factor loadings, uniqueness, and CITC scores for the 15-Item FRQ.

Item	Statistic		
	Factor loading	Uniqueness	CITC
2. I am treated like a human being here	**0.690**	**0.531**	**0.715**
4. I can express my feelings here enough	**0.724**	**0.476**	**0.733**
7. The hospital helps me practice hobbies I like	**0.627**	**0.607**	**0.617**
9. I feel included in my care plan enough (CPA and Ward Rounds)	**0.706**	**0.501**	**0.761**
10. I am given enough information about my care	**0.679**	**0.539**	**0.683**
16. Staff respect me as an individual	**0.662**	**0.562**	**0.694**
21. I am given enough responsibility on the ward	**0.664**	**0.559**	**0.676**
22. I am trusted by staff enough	**0.620**	**0.616**	**0.621**
25. I can choose what I want to do each day	**0.652**	**0.575**	**0.631**
28. It is fair I am here right now	**0.622**	**0.613**	**0.580**
29. I can participate in activities I find meaningful	**0.641**	**0.589**	**0.627**
46. My rights are respected properly here	**0.724**	**0.476**	**0.708**
49. I am forced to do things I don’t want to do	**0.630**	**0.604**	**0.532**
54. The rules on the ward are fair	**0.716**	**0.488**	**0.676**
55. The restrictions on the ward make sense	**0.658**	**0.673**	**0.568**

CITC, Corrected Item Total Correlation; FRQ, Forensic Restrictiveness Questionnaire.

### Reliability

The resulting FRQ scale was highly internally consistent. Cronbach’s α = 0.93. CITC scores ranged from α = 0.53 to 0.76. These are presented in Table. This suggests the FRQ was internally reliable as hypothesized.

### Convergent Validity

The directions and significance of the associations were as hypothesized. The FRQ correlated negatively with the EssenCES total score (Spearman’s *ρ* = −0.61, p < .001, n = 229, *R* *^2^* = .372). There was a negative correlation between the FRQ and the FQL-SV (Spearman’s *ρ* = −0.72, p < .001, n = 229, *R* *^2^* = .518). The EssenCES and FQL-SV correlated significantly in a positive direction (Spearman’s *ρ* = 0.57, p < .001, n = 229). These associations are classed as moderate to strong (49). These results and correlations with EssenCES domains are presented in **Table 4**.

**Table 4:** Spearman correlations between FRQ, FQL-SV, and EssenCES (and domains).

	FRQ	EssenCES	FQL-SV
**EssenCES**	−0.61		
**FQL-SV**	−0.72	0.58	
***Patient Cohesion***	−0.35	0.77	0.43
***Experienced Safety***	−0.39	0.62	0.27
***Therapeutic Hold***	−0.63	0.73	0.58

All results p < 0.001; n = 229; The sub-domains of EssenCES are italicized.

EssenCES, Essen Climate Evaluation Schema; FQL-SV, Forensic Quality of Life Profile-Short Version; FRQ, Forensic Restrictiveness Questionnaire.

### Recent Hard, Difficult, or Hurtful Events

Patients that expressed experiencing something very hard, difficult, or hurtful in the week prior to completing the FRQ (Mean rank = 150.68, n = 64) scored significantly higher than those individuals not reporting this (Mean rank = 105.54, n = 172), U = 3275.0, p < .001, r = −.29.

## Discussion

Forensic in-patient services aim to provide care in secure, restrictive settings. Therapeutic ideals promoting autonomy and patient-involvement can clash with custodial prerogatives (54). The nature of these restrictions can have significant impacts upon patient recovery. Such restrictions can be counter to human rights ideals (55), best practices, and contemporary models of rehabilitation such as the recovery approach (17) and the Good Lives Model (56). Accordingly, a measure of patient experiences of these restrictions is warranted.

The present study described such a measure: the FRQ. A pool of items was developed from qualitative interviews with patients in low, medium, and high secure settings (see 29). These items were submitted to a panel of experts in the field of forensic psychiatry and revised. A 56-item FRQ was piloted with 241 patients across secure hospitals in England. The results of a psychometric analysis indicate that the FRQ has unidimensional structure, captured by 15 items. The FRQ correlated negatively with measures of Quality of Life and Ward Atmosphere as hypothesized and was found to be internally consistent.

The FRQ was strongly correlated with quality of life. Increasing attention is paid to the role QoL plays in patient mental health. This is evidenced in contemporary models of offender rehabilitation. These include the Good Lives Model (56) and the application of recovery principles to forensic settings (14–16). These approaches prioritize strength-building and emphasize quality of life.

Quality of life is both a predictor and outcome in forensic services. QoL is generally acknowledged as a key indicator of clinical mental well-being (57). As a predictor, Bouman et al. (58) demonstrated that in a forensic out-patient context higher levels of satisfaction with one’s quality of life and one’s health were associated with lower recidivism rates. As an outcome measure, QoL has been predicted by a range of psychosocial variables in forensic settings. Of relevance for this study, Long et al. (59) reported that level of security, as well as psychopathology and living conditions, was significantly associated with QoL scores. The authors attribute this to the degree of control and mastery patients have over their own lifestyle. Further, O’ Flynn and others (57) found that level of security, availability of meaningful activity, and TH between staff and patients were significant predictors of total QoL scores.

The FRQ includes questions on patient control and choice, access to meaningful activities, and on restrictions more generally. Thus, given the relationships between restrictiveness and QoL, taking seriously patients’ accounts of restrictiveness as captured in the FRQ and incorporating this into routine care might be significant in improving patient QoL and other outcomes.

The FRQ was also strongly correlated with ward atmosphere. Closed and restrictive atmospheres characterized by stress, fear, and inflexibility have been associated with negative emotions, hostility, anti-social behavior, low social engagement, and increased verbal, and physical aggression (60–62).

Social climate of forensic settings has been shown to predict reoffending. A recent study explored the predictive ability of prison social climate on proven reoffending within 12 months of release (63). A multilevel regression model controlling for security level, inmate age, inmate ethnicity, and percentage of prisoners completing an offending behavior program found that prisoner adaptation, drugs, bullying, exploitation, safety, staff supervision and control, and individual autonomy most strongly predicted reoffending.

Given the association between the FRQ and EssenCES, interventions to reduce untherapeutic restrictions might foster a more open and positive ward atmosphere. This could have positive consequences on patient outcomes and improve conditions for staff and patients.

The correlations between the FRQ and measures of QoL and ward atmosphere ask us to consider to what extent restrictiveness so conceived is a distinct construct from or a proxy of these or a third variable, such as satisfaction with care. Empirically, the amount of shared variance between the FRQ and FQL-SV (52%) and the EssenCES (37%) suggests that these constructs do overlap. This overlap might be due to a shared focus on autonomy or patients’ use of these measures as a proxy for general dissatisfaction in their care. Much variance is not shared however. The explanation for this may be conceptual. Restrictiveness diverges from QoL and ward atmosphere as it aims to capture restrictions on patients’ sense of self/identity and personhood as well as the degree to which restrictions are fair or make sense to them. The FRQ can therefore complement not supplement these other measures.

The outcomes of this study add to the findings of Franke et al. (31). Measuring perceived restraint with the aMQPL in German secure settings, the authors found that scores were associated with psychological symptoms including hostility, depression, and psychological state more broadly in a negative direction. High perceived restraint scores were also associated with a higher likelihood of suicidal ideation. These studies suggest that, though complex and the direction of causality unclear, the relationship between patients’ experiences of restrictiveness and adverse therapeutic processes and outcomes cannot be ignored and deserves further clinical and scientific attention.

## Limitations

This study has a number of shortcomings. Random sampling was not employed. As participation was voluntary and consensual, only individuals who had an interest, were not in seclusion and had capacity to consent were involved. Participation may have appealed to patients with strong feelings on the topic. Further studies should explore the discriminant validity of the FRQ; specifically, its relationship with constructs such as general satisfaction with life or care. Given the higher scores reported by patients having experienced something they consider very hard, difficult, or hurtful in the week prior to completing the FRQ, it is plausible that responses on the FRQ reflect patients’ dissatisfaction with care more generally. These studies could include a validated forensic measures such as the Forensic Satisfaction Survey (64).

Female patients were underrepresented as they comprised 4% of the current sample but are approximately 12% of the forensic population (65). These factors might have biased the responses on the FRQ and rendered the results less generalizable.

The sample size (N = 235) was comparable to similar studies developing questionnaires in forensic settings (E.G., 46, 64) but fell short of ideal participant to item ratios for factor analysis as recommended in the literature, I.E., 10:1 (52, 66). Further replicative studies should aim for a larger and more representative sample with more participants to ensure a more accurate distribution of all patient groups, including those diagnosed with a learning disability. It is a further limitation that the resulting 15-Item FRQ has only one reverse-coded item; this reduces the possibility to detect some response biases (e.g., fatigue or yea-/nay-saying).

## Implications of This Project

The FRQ has clinical value; it can provide a springboard for care staff to discuss specific elements of care patients wish to describe based on their answers to each of the FRQ items. This interviewing could be part of patients’ care plans. This proactive and inclusive approach to care planning is integral to the ethos of patient-centered care, independence, and shared decision-making (7–11).

The FRQ has scientific value. Studies could explore causality between restrictiveness, ward atmosphere, and quality of life by employing repeated measures and conducting an analysis of variance over time controlling for possible confounding variables such as ward-type, level of security, medications, treatment and recovery outcomes, diagnosis, patient profiles, and recent adverse events. Differences in mean FRQ scores could be compared between clinical and demographic groups. The FRQ offers opportunities for ward, hospital, and international comparisons. Following the presentation of the preliminary results of this study plans are underway to validate the FRQ in Canada, Germany, Poland, and Italy.

Further, the FRQ could be used as a measure of change following alterations in local treatment philosophy, service reorganization, or the introduction of initiatives to reduce restrictions. Prior to being used in this way however, the sensitivity to change of the FRQ needs to be established. Future projects should investigate sensitivity to change. The FRQ can be accessed at: www.frqquestionnaire.weebly.com or by asking the corresponding author.

## Conclusion

The 56-item FRQ was completed by a sample of 235 patients from 16 NHS Trusts in England. These patients resided in low, medium, and high secure forensic settings across England. Patients with a range of demographic, clinical, and legal backgrounds participated. The findings of the psychometric investigations suggested that a unidimensional structure was the most adequate for explaining a meaningful proportion of variance in FRQ scores. The short, 15-item final FRQ was highly internally consistent. The final FRQ correlated with measures of ward climate and quality of life in the hypothesized directions, thus placing the FRQ within a nomothetic network and providing empirical evidence supporting claims of construct validity. The FRQ offers a novel and helpful method for clinicians and researchers to measure and explore forensic patients’ experiences of restrictiveness within secure hospitals.

## Data Availability Statement

The datasets generated for this study will not be made publicly available. Ethical approval was not given for sharing the raw datasets given the patient population involved in this study.

## Ethics Statement

The studies involving human participants were reviewed and approved by NHS Health Research Authority; Leicestershire South Research Ethics Committee. Study Number: 17/EM/0159. The patients/participants provided their written informed consent to participate in this study.

## Author Contributions

JT, BV, PB, and VE contributed to the conception and design of the study. JT and VF collected data. JT and VE performed the quantitative analysis. JT wrote the first draft of the manuscript. All authors approved the submitted version.

## Funding

This work was funded by the Economic and Social Research Council (ESRC) (Grant Number ES/J500100/1).

## Conflict of Interest

The authors declare that the research was conducted in the absence of any commercial or financial relationships that could be construed as a potential conflict of interest.

## References

[1] Chow WS Priebe S How has the extent of institutional mental healthcare changed in Western Europe? Analysis of data since 1990. BMJ Open (2016) 6:10188. 10.1136/bmjopen-2015 PMC4854016 27130161

[2] Goffman E Asylums: essays on the social situation of mental patients and other inmates. Aldine Transaction (1961) London, UK.

[3] Tomlin J Bartlett P Völlm B Experiences of restrictiveness in forensic psychiatric care: Systematic review and concept analysis. Int J Law Psychiatry (2018) 57:31–41. 10.1016/j.ijlp.2017.12.006 29548502

[4] Salize HJ Dreßing H Peitz M Compulsory admission and involuntary treatment of mentally ill patients-legislation and practice in EU-member states. Mannheim, Germany: Central Institute of Mental Health Research Project Final Report (2002). 15 p.

[5] Care Quality Commission (2018). The state of care in mental health services 2014 to 2017 | Care Quality Commission 4 https://doi.org/CQC-380-072017.

[6] DoH (2015). Mental Health Act 1983: Code of Practice. Retrieved from 5. www.tsoshop.co.uk.

[7] JCPMH (2013). Guidance for commissioners of forensic mental health services: Joint Commissioning Panel on Mental Health Retrieved from www.jcpmh.info.

[8] NHS England (2018). Service specification: low secure mental health services (Adult). Retrieved from https://www.england.nhs.uk/publication/service-specification-low-secure-mental-health-services-adult/.

[9] NICE (2011). Service user experience in adult mental health services: Quality standard. Retrieved from https://www.nice.org.uk/guidance/qs14.

[10] The Mental Health Taskforce (2016). The Five Year Forward View for Mental Health. Retrieved from https://www.england.nhs.uk/wp-content/uploads/2016/02/Mental-Health-Taskforce-FYFV-final.pdf.

[11] Wessely S Modernising the Mental Health Act: Increasing choice, reducing compulsion. In: Final report of the Independent Review of the Mental Health Act 1983. (2018). Retrieved from www.gov.uk/dhsc.

[12] Caldas-Almeida J Mateus P& Gina T (2016). Towards community-based and socially inclusive mental health care. Joint Action on Mental Health and Well-being. Situation analysis and recommendations for action. Retrieved from 12. https://ec.europa.eu/health/sites/health/files/mental_health/docs/2017_towardsmhcare_en.pdf.

[13] Council of Europe (2004). Reccomendation No. REC(2004)10 of the Committee of Ministers to member States concerning the protection of the human rights and dignity of persons with mental disorder and its Explanatory Memorandum.

[14] Drennan G Wooldridge J Aiyegbusi A Alred D Ayres J Barker R Making Recovery a Reality in Forensic Settings. (2014) 1–28. Retrieved from https://www.nhsconfed.org/-/media/Confederation/Files/Publications/Documents/making-recovery-reality-forensic-settings.pdf.

[15] Mann B Matias E Allen J Recovery in forensic services: facing the challenge. Adv Psychiatr Treat (2014) 20(2):125–31. 10.1192/apt.bp.113.011403

[16] Simpson AIF Penney SR Recovery and forensic care: Recent advances and future directions. Crim Behav Ment Health (2018) 28:383–9. 10.1002/cbm.2090 30215871

[17] Jacobson N Greenley D What Is Recovery? A Conceptual Model and Explication. Psychiatr Serv (2001) 52(4):482–5. 10.1176/appi.ps.52.4.482 11274493

[18] Leamy M Bird V Le Boutillier C Williams J Slade M Conceptual framework for personal recovery in mental health: systematic review and narrative synthesis. Br J Psychiatry (2011) 199(6):445–52. 10.1192/bjp.bp.110.083733 22130746

[19] Clarke C Lumbard D Sambrook S Kerr K What does recovery mean to a forensic mental health patient? A systematic review and narrative synthesis of the qualitative literature. J Forensic Psychiatry Psychol (2016) 27(1):38–54. 10.1080/14789949.2015.1102311

[20] Ewington J Best practices for reducing the use of coercive measures. In The Use of Coercive Measures in Forensic Psychiatric Care. Springer, Cham (2016). 285–314 p.

[21] Adshead G Davies T Wise restraints: Ethical issues in the coercion of forensic patients. In: The Use of Coercive Measures in Forensic Psychiatric Care: Legal, Ethical and Practical Challenges. Switzerland: Springer International Publishing (2016). 69–86 p. 10.1007/978-3-319-26748-7_5

[22] Elcock S Lewis J Mechanical restraint: Legal, ethical and clinical issues. In: The Use of Coercive Measures in Forensic Psychiatric Care: Legal, Ethical and Practical Challenges. Switzerland: Springer International Publishing (2016). 315–31 p. 10.1007/978-3-319-26748-7_17

[23] Hui A Middleton H Vollm B The uses of coercive measures in forensic psychiatry: A literature review. In: The Use of Coercive Measures in Forensic Psychiatric Care: Legal, Ethical and Practical Challenges. Switzerland: Springer International Publishing (2016). 151–84 p.

[24] Newton-Howes G Banks D The subjective experience of community treatment orders: patients’ views and clinical correlations. Int J Soc Psychiatry (2014) 60(5):474–81. 10.1177/0020764013498870 23985374

[25] Soininen P Kontio R Joffe G Putkonen H Patient experience of coercive measures. In: The Use of Coercive Measures in Forensic Psychiatric Care: Legal, Ethical and Practical Challenges. (2016). 255–70 p. 10.1007/978-3-319-26748-7_14

[26] Kontio R Joffe G Putkonen H Kuosmanen L Hane K Holi M Seclusion and restraint in psychiatry: patients’ experiences and practical suggestions on how to improve practices and use alternatives. Perspect Psychiatr Care (2012) 48(1):16–24. 10.1111/j.1744-6163.2010.00301.x 22188043

[27] Sustere E Tarpey E Least restrictive practice: its role in patient independence and recovery. J Forensic Psychiatry Psychol (2019) 30(4):614–29. 10.1080/14789949.2019.1566489

[28] Hui A Least restrictive practices: an evaluation of patient experiences. University of Nottingham (2017). Retrieved from http://eprints.nottingham.ac.uk/48816/.

[29] Tomlin J. Egan V. Bartlett P. Völlm B. (2019). What Do Patients Find Restrictive About Forensic Mental Health Services? A Qualitative Study. International Journal of Forensic Mental Health, 1–13.

[30] Sexton L Penal subjectivities: Developing a theoretical framework for penal consciousness. Punishment Soc (2015) 17(1):114–36. 10.1177/1462474514548790

[31] Franke I Buesselmann M Streb J Dudeck M Perceived institutional restraint is associated with psychological distress in forensic psychiatric inpatients. Front Psychiatry 10:410. 31244698 10.3389/fpsyt.2019.00410 PMC6580144

[32] Høyer G Kjellin L Engberg M Kaltiala-Heino R Nilstun T Sigurjónsdóttir M Paternalism and autonomy: a presentation of a Nordic study on the use of coercion in the mental health care system. Int J Law Psychiatry (2002) 24(2):93–108. 10.1016/S0160-2527(01)00108-X 12071105

[33] Bergk J Flammer E Steinert T Coercion Experience Scale (CES) - validation of a questionnaire on coercive measures. BMC Psychiatry (2010) 10(1):5. 10.1186/1471-244x-10-5 20074355 PMC2837616

[34] Adcock R Collier D Measurement validity: a shared standard for qualitative and quantitative research. Am Polit Sci Rev (2001) 95(3):529–46.

[35] Luyt R A framework for mixing methods in quantitative measurement development, validation, and revision: a case study. J Mixed Methods Res (2012) 6(4):294–316.

[36] Messik S Validity of psychological assessment. Am Psychol (1995) 50(9):741–9.

[37] Cooper C (2018). Psychological testing: theory and practice. Retrieved from https://books.google.co.uk/books?hl=en&lr=&id=XmNwDwAAQBAJ&oi=fnd&pg=PT19&dq=Psychological+testing:+Theory+and+Practice+Colin+Cooper&ots=1ITieM01an&sig=6kBUKqSgG3ktIdIGm8Wzkkyh8zMv=onepage&q=Psychologicaltesting%3ATheoryandPracticeColinCooper&f=fal.

[38] Hinkin TR A brief tutorial on the development of measures for use in survey questionnaires. Organ Res Methods (1998) 1(1):104–21. 10.1177%2F109442819800100106

[39] Streiner DL Norman GR Health Measurement Scales. Oxford University Press (2008) Oxford, UK. 10.1093/acprof:oso/9780199231881.001.0001

[40] Creswell JW Clark VLP Designing and conducting mixed methods research. Thousand Oaks, CA, US: Sage Publications, Inc. (2007).

[41] Lynn P Principles of Sampling. In: Greenfield T Greener S, editors. Research Methods for Postgraduates. London, UK: Wiley (2016). Retrieved from https://ebookcentral.proquest.com/lib/nottingham/reader.action?docid=4644084&ppg=268.

[42] Silverman D Interpreting qualitative data. Sage (2015) London, UK.

[43] Schalast N Redies M Collins M Stacey J Howells K EssenCES, a short questionnaire for assessing the social climate of forensic psychiatric wards. Crim Behav Ment Health (2008) 18(1):49–58. 10.1002/cbm.677 18229876

[44] Milsom SA Freestone M Duller R Bouman M Taylor C Factor structure of the Essen Climate Evaluation Schema measure of social climate in a UK medium-security setting. Crim Behav Ment Health (2014) 24(2):86–99. 10.1002/cbm.1878 23996523

[45] Schel SHH Bouman YHA Vorstenbosch ECW Bulten BH Development of the forensic inpatient quality of life questionnaire: short version (FQL-SV). Qual Life Res (2017) 26(5):1153–61. 10.1007/s11136-016-1461-9 27878427

[46] Vorstenbosch EC Bouman YH Braun PC Bulten EB Psychometric properties of the forensic inpatient quality of life questionnaire: quality of life assessment for long-term forensic psychiatric care. Health Psychol Behav Med (2014) 2(1):335–48. 10.1080/21642850.2014.894890 PMC4346075 25750786

[47] Furr R Scale Construction and Psychometrics for Social and Personality Psychology. SAGE (2011) London, UK SAGE. 104135/9781446287866

[48] Osborne JW Costello AB Kellow JT (2008). Best practices in xploratory factor analysis. Best practices in quantitative methods, 86–99.

[49] Dancey CP Reidy J Statistics Without Maths for Psychology: Using Spss for Windows. (UK): Prentice Hall (2014). p. 619 Retrieved from http://catalogue.pearsoned.co.uk/educator/product/Statistics-Without-Maths-for-Psychology/9780273774990.page.

[50] Fabrigar LR Wegener DT MacCallum RC Strahan EJ Evaluating the use of exploratory factor analysis in psychological research. Psychol Methods (1999) 4(3):272–99. 10.1037/1082-989X.4.3.272

[51] Andy F Discovering statistics using SPSS. London: UK (2009).

[52] Tabachnick BG Fidell LS Using Multivariate Statistics. Harper Collins. New Jersey: Pearson (2013). 10.1037/022267

[53] Flesch R How to write plain English: a book for lawyers and consumers (pp. 20–32). New York, NY: Harper & Row (1979).

[54] Adshead G Care or custody? Ethical dilemmas in forensic psychiatry. J Med Ethics (2000) 26(5):302–4. 10.1136/jme.26.5.302 PMC1733273 11055029

[55] Albrecht HJ Legal aspects of the use of coercive measures in psychiatry. The use of coercive measures in forensic psychiatric care: legal, ethical and practical challenges. (2016) 31–48. 10.1007/978-3-319-26748-7_3

[56] Ward T Brown M The good lives model and conceptual issues in offender rehabilitation. Psychol Crime Law (2004) 10(3):243–57.

[57] O’Flynn P O’Regan R O’Reilly K Kennedy HG Predictors of quality of life among inpatients in forensic mental health: implications for occupational therapists. BMC Psychiatry (2018) 18(1):16. 29351784 10.1186/s12888-018-1605-2 PMC5775562

[58] Bouman YH Schene AH de Ruiter C Subjective well-being and recidivism in forensic psychiatric outpatients. Int J Forensic Ment Health (2009) 8(4):225–34.

[59] Long C McLean A Boothby A Hollin C Factors associated with quality of life in a cohort of forensic psychiatric in-patients. Br J Forensic Pract (2008) 10(1):4–11.

[60] Dickens GL Suesse M Snyman P Picchioni M Associations between ward climate and patient characteristics in a secure forensic mental health service. J Forensic Psychiatry and Psychol (2014) 25(2):195–211.

[61] Tonkin M A review of questionnaire measures for assessing the social climate in prisons and forensic psychiatric hospitals. Int J Offender Ther Comp Criminol (2016) 60(12):1376–405. 10.1177/0306624X15578834 25850103

[62] Van der Helm P Stams GJ Van der Laan P Measuring group climate in prison. Prison J (2011) 91(2):158–76.

[63] Auty KM Liebling A Exploring the relationship between prison social climate and reoffending. Justice Q (2019) 1–24.

[64] MacInnes D Beer D Keeble P Rees D Reid L The development of a tool to measure service user satisfaction with in-patient forensic services: the forensic satisfaction scale. J Ment Health (Abingdon England) (2010) 19(3):272–81. 10.3109/09638231003728133 20441491

[65] Rutherford M Duggan S Forensic mental health services: facts and figures on current provision. Br J Forensic Pract (2008) 10(4):4–10.

[66] MacCallum RC Widaman KF Zhang S Hong S Sample size in factor analysis. Psychol Methods (1999) 4(1):84.

