# Corrigendum: The Forensic Restrictiveness Questionnaire: Development, Validation, and Revision

**DOI:** 10.3389/fpsyt.2020.00128

**Published:** 2020-02-25

**Authors:** Jack Tomlin, Birgit Völlm, Vivek Furtado, Vincent Egan, Peter Bartlett

**Affiliations:** ^1^Department of Forensic Psychiatry, University of Rostock, Rostock, Germany; ^2^Mental Health and Wellbeing, Warwick Medical School, University of Warwick, Coventry, United Kingdom; ^3^Centre for Family and Forensic Psychology, University of Nottingham, Nottingham, United Kingdom; ^4^School of Law and Institute of Mental Health, University of Nottingham, Nottingham, United Kingdom

**Keywords:** forensic, mental health, restrictive, autonomy, FRQ, forensic restrictiveness questionnaire, psychometric

In the original article, there was an error. The Forensic Inpatient Quality of Life Questionnaire - Short Version (FQL-SV) was incorrectly called the Forensic Quality of Life Profile - Short Version (FQL-SV).

A correction has been made to the **Instruments** section, subsection Forensic Quality of Life Profile-Short Version (FQL-SV), Paragraph 1:

“**Forensic Inpatient Quality of Life Questionnaire - Short Version (FQL-SV)**

Patient quality of life was measured with the short version of the Forensic Inpatient Quality of Life Questionnaire - Short Version (FQL-SV; 45, 46). This scale was developed in The Netherlands and translated into English by its authors. The FQL-SV is comprised of 20 items. It asks patients about a range of topics including leave, safety, food, personal hygiene, sexuality, and relationships with other residents.”

The authors apologize for this error and state that this does not change the scientific conclusions of the article in any way. The original article has been updated.

